# Results after one year introduction of disease oriented payments in the Netherlands

**Published:** 2012-09-04

**Authors:** Christel E van Dijk, Ilse C.S Swinkels, Mieke Rijken, J Korevaar, Dinny H de Bakker

**Affiliations:** NIVEL, Netherlands Institute for Health Services Research, Utrecht, The Netherlands; NIVEL, Netherlands Institute for Health Services Research, Utrecht, The Netherlands; NIVEL, Netherlands Institute for Health Services Research, Utrecht, The Netherlands; NIVEL, Netherlands Institute for Health Services Research, Utrecht, The Netherlands; NIVEL, Netherlands Institute for Health Services Research, Utrecht, The Netherlands and Tilburg University, Scientific Centre for Transformation in Care and Welfare (TRANZO), Tilburg, The Netherlands

**Keywords:** disease oriented payments, chronically ill, diabetes mellitus type II, the Netherlands

## Abstract

**Purpose:**

The purpose of our study was to show the first results after introduction of disease oriented payments for diabetes mellitus type II in the Netherlands.

**Theory:**

In 2010 disease oriented payment for diabetes mellitus type II was introduced nationwide in the Netherlands. In disease oriented payment, care for disease groups is organised by a care group that organises both general and more specialised care, and that negotiate a lumpsum for each patient with the health insurer. The care groups can either provide care themselves or sub-contract other providers. Included services within the care program are based on national health care standards. Aim of disease oriented payments is to improve care for chronically ill, by stimulating multidisciplinary collaboration between health care providers.

**Methods:**

Selection of patients, health care utilization, organisation of care and self management needs were analysed with the aid data from written structured questionnaires from the National Panel of people with Chronic Illness or Disability (n=275) and data from electronic medical records of general practitioners (n=1144). Diabetes patients with and without disease oriented payment were compared.

**Results and conclusions:**

Our first results show no evidence of selection of patients within disease oriented payments. And hardly any differences in the care between diabetes patients with and without disease oriented payments. Diabetes patients within disease oriented payments tended to go less often to a dietician after the introduction of disease oriented payments. It seems that patients within disease oriented payments receive part of their care from less specialist health care providers. Also, care to diabetes patients within disease oriented payment was only to a limited degree provided according to a programmatic approach.

Powerpoint presentation available at http://www.integratedcare.org at congresses – San Marino – programme.

